# A novel approach toward skin cancer classification through fused deep features and neutrosophic environment

**DOI:** 10.3389/fpubh.2023.1123581

**Published:** 2023-04-17

**Authors:** Ahmed Abdelhafeez, Hoda K. Mohamed, Ali Maher, Nariman A. Khalil

**Affiliations:** ^1^Faculty of Information Systems and Computer Science, October 6th University, Cairo, Egypt; ^2^Faculty of Engineering, Ain Shams University, Cairo, Egypt; ^3^Military Technical College, Cairo, Egypt

**Keywords:** transfer learning, fused deep features, skin cancer classification, multi-support vector machine, error-correcting output codes, single-valued neutrosophic sets

## Abstract

Variations in the size and texture of melanoma make the classification procedure more complex in a computer-aided diagnostic (CAD) system. The research proposes an innovative hybrid deep learning-based layer-fusion and neutrosophic-set technique for identifying skin lesions. The off-the-shelf networks are examined to categorize eight types of skin lesions using transfer learning on International Skin Imaging Collaboration (ISIC) 2019 skin lesion datasets. The top two networks, which are GoogleNet and DarkNet, achieved an accuracy of 77.41 and 82.42%, respectively. The proposed method works in two successive stages: first, boosting the classification accuracy of the trained networks individually. A suggested feature fusion methodology is applied to enrich the extracted features’ descriptive power, which promotes the accuracy to 79.2 and 84.5%, respectively. The second stage explores how to combine these networks for further improvement. The error-correcting output codes (ECOC) paradigm is utilized for constructing a set of well-trained true and false support vector machine (SVM) classifiers *via* fused DarkNet and GoogleNet feature maps, respectively. The ECOC’s coding matrices are designed to train each true classifier and its opponent in a one-versus-other fashion. Consequently, contradictions between true and false classifiers in terms of their classification scores create an ambiguity zone quantified by the indeterminacy set. Recent neutrosophic techniques resolve this ambiguity to tilt the balance toward the correct skin cancer class. As a result, the classification score is increased to 85.74%, outperforming the recent proposals by an obvious step. The trained models alongside the implementation of the proposed single-valued neutrosophic sets (SVNSs) will be publicly available for aiding relevant research fields.

## 1. Introduction

Skin cancer is common throughout the world, and it is responsible for many fatalities each year (1). Because it is such an aggressive disease, early discovery is critical to preserve lives. Skin cancer cases are increasing in both developed and developing countries. Skin cancer cases increased to 1.2 million in 2020, and according to the World Health Organization (WHO), there will be approximately 2.2 million cancer cases by 2025 (2). Early tumor detection and classification of malignant and benign tumors, in contrast, have a considerable effect on survival (3). According to recent research, early diagnosis raised 5-year survival rates to 91% (4). This is exacerbated by a global shortage of radiologists and doctors capable of interpreting screening data, particularly in rural areas and developing nations (5). Humanoid resources and technologies to give quick patient care by using screening, diagnosis, and treatment are vital because time is a major factor in saving lives (6).

However, due to the presence of noise, artifacts, and complicated structures, tumor detection takes time and is often difficult for radiologists who review medical pictures. Furthermore, an increasing count of lesions adds to the radiologist’s workload, which frequently leads to tumor misdiagnosis and can lead to poor tumor detection performance. When compared to the human eye, dermoscopy is a common skin imaging technique that has been utilized for building benchmark datasets to improve melanoma diagnosis (7). Few forms of skin cancer are included in the majority of these small datasets, and there may not be many images in any class. In addition, three crucial factors reduce the accuracy of automated melanoma detection using dermoscopy images. First, while skin lesions are divided into various groups, their characteristics, such as size, texture, color, and form, are quite similar, making classification difficult. Second, melanoma and non-melanoma lesions have a significant relationship. The environment, which includes hair, veins, and illumination, is the third factor (8). When the number of images required to train a deep convolutional neural network (DCNN) is insufficient, conventional augmentation is widely used. However, publicly available skin cancer databases are severely unbalanced, with an unfair instance count for each class. For technological research, ISIC created the ISIC Archive, a global library of dermoscopy images.^1^

The proposed study spots the light on that unfairness to fairly augment leaked-instance classes. In the medical area, transfer learning (TL) is critical for improving diagnosis performance, particularly while dealing with the multifaceted properties of skin cancer images. Scholars have recently become interested in fine-tuned TL networks with pre-trained weights for performing complex classification tasks with substantial interpretation performance. The proposed study adopts TL to fine-tune the well-known deep networks for investigating the top ones in the skin cancer classification task. Image processing and machine learning approaches may be useful for detection and diagnosis, but they frequently result in false-positive and false-negative cases (9). There are several deep learning procedures obtainable right now, but not all of them have been tested for their effectiveness in identifying skin cancer. Such algorithms extract essential distinguishing features from images without the need for manual human intervention, allowing for fully automatic mass segmentation, discovery, and organization. When the number of images required to train a deep convolutional neural network (DCNN) is insufficient, the augmentation technique can be applied to have a sufficient number of images.

Several studies created binary or multiclass skin cancer classification models, but they were unable to determine which model was best. Individual models have been used for binary and multiclass skin cancer classification, with varying degrees of success, including CNN-PA (10), EfficentNet (11), MobileNet (12), VGGNET (13), AlexNet (14), GoogleNet (15), and LCNET (16). A model selection experiment must be carried out to fairly assess which model is preferable for the same dataset and number of classes. In the first planned experiment, five models have been examined to determine which offers a more useful option and a more reliable decision. The accuracy of each model has been independently increased by utilizing the feature fusion method to further construct the two superior models. The two best models for the second planned experiment are produced by using such models.

In the study, ensemble models outperformed individual deep learners in performance ratios, while dermatologists’ diagnosis accuracy classification outperformed both. A machine learning technique called ensemble combines the judgments of multiple individual learners to improve classification accuracy (17). It is anticipated that the ensemble model will improve classification accuracy since it draws on the diversity of the individual models to form a collective judgment. In many articles, researchers employed different ensemble models to determine which combination was best. In Ref. (18), GoogleNet, AlexNet, and VGGNet were combined to achieve the desired outcome, while in Ref. (19), GoogleNet, AlexNet, and VGGNet were combined to achieve various outcomes. It was unclear why certain combinations of models would produce outcomes with higher accuracy, and what if choosing a different combination would produce the best outcome? Depending on the outcome of the first planned experiment, the top two models, DarkNet and GoogleNet, have been fused in the second proposed experiment to obtain a suitable fusion selection rather than a random one.

Neutrosophic approaches have recently been proposed by researchers to improve classification performance. An intelligent neutrosophic diagnostic method for cardiotocography data was suggested by the authors in Ref. (20). These techniques demonstrate that some lesions can be neutrosophically categorized even when individual or combination models fail to yield the desired classification. A brand-new method for multiclass skin cancer categorization was created. According to experimental findings, adding the single-valued neutrosophic sets (SVNSs) to the combined deep learners for feature fusion methodology (FFM) results in increased skin cancer classification accuracy. The findings indicate that the suggested models perform better than the multiclass skin cancer classification models that have recently been created.

The following contributions are made to this research work:

- To deal with over-fitting difficulties, the skin lesion dataset is categorized tacitly into eight classes in terms of the number of their instances which is rich and poor clusters. Various spatial data augmentation methods are applied to the poor cluster to balance the dataset on the fly in the training phase.
- To find the superior net model for the ISIC 2019 dataset, pre-trained networks such as DarkNet, AlexNet, GoogleNet, XceptionNet, and DenseNet are examined using transfer learning.
- After extensive research, a feature fusion methodology is suggested for the top two models. The fusion is carried for enriching the feature descriptive supremacy by fusing different feature maps from various network layers.
- To exploit the individual descriptive power for each net fused feature, a set of true and false classifiers is designed and trained *via* the ECOC scheme.
- Each true classifier and its opponent may disagree with the classification result, which establishes an ambiguity. This ambiguity is quantified by an indeterminacy set and resolved by the SVNSs to further investigate the robustness.

Furthermore, it is worth noting that the work is made generally where it is applicable for boosting any individual classifiers and resolving their contradictions and dependably that no pre-processing of the images or lesion segmentation has been done.

The rest of this article is organized as follows: Section 2 provides the materials and methods. Section 3 describes the experimental results. A discussion is presented in Section 4. The conclusion of this article and future studies are presented in Section 5.

**Figure 1:**
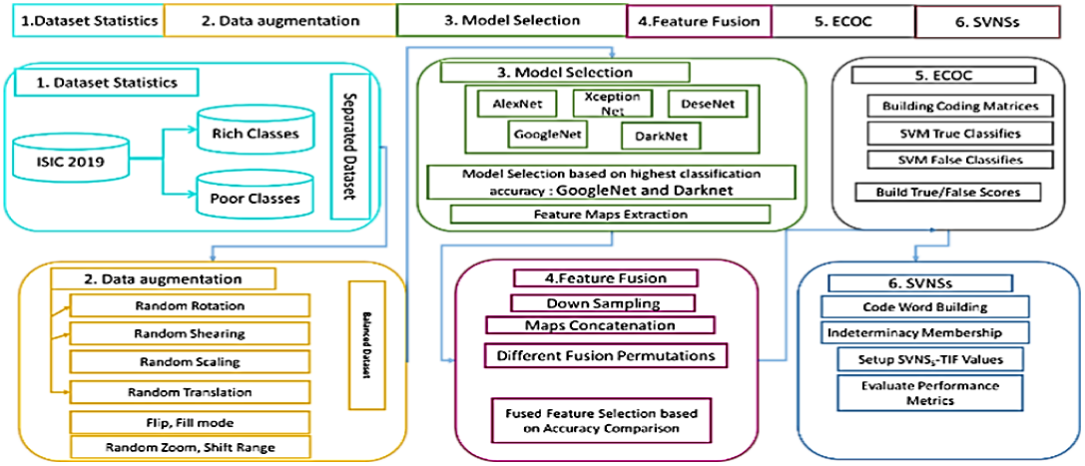
Full technique of skin cancer categorization described in this article.

Figure 1 depicts the whole categorization process as a series of blocks. The original ISIC 2019 skin cancer dataset is represented by block (1). A dataset has been supplemented using data augmentation methods on a pre-processed dataset (2). Experimenting with a variety of pre-trained models and choosing the most accurate model for use with an updated dataset yields suggested GoogleNet and DarkNet models (3). After a suggested model was selected, a feature fusion method was applied based on accuracy comparison (4). The ECOC’s coding matrices are designed to train each classifier and its opponent in one-versus-another fashion (5) and (6) the final model was assessed using SVNSs to get superior accuracy.

## 2. Materials and equipment

Several artificial intelligence-based techniques have been developed to automate the classification process, which includes standard phases, such as pre-processing, feature extraction, segmentation, and classification. Many classification methods rely heavily on constructed feature sets, which have limited generalizability for dermoscopic skin pictures due to a lack of biological pattern knowledge (21). Because of their closeness in size, shape, and colors, lesions have a strong visual likeness and are highly linked, resulting in poor feature information (22). Handmade, feature-based methods are, therefore, useless for skin classification. Deep networks are more effective in calculating specific features to do precise lesion categorization than shallow networks. Convolutional neural networks (CNNs) are widely used for medical image processing and categorization. Authors in Ref. (10) made the first breakthrough in using DCNN for skin cancer. To make a binary classification between the two fatal skin tumors, malignant and nevus, the network was likened to 21 board-certified medicinal professionals. Specialists testified that the envisioned network could accurately detect skin cancer. The dataset of 1,29,450 clinical images, including 3,374 dermoscopic images, was processed using the InceptionV3 architecture pre-trained on ImageNet. The scientists demonstrated that a deep neural network-based solution outperformed clinical professionals in terms of dermoscopy picture categorization accuracy over a large dataset. It is demonstrated that artificial intelligence can diagnose skin cancer with a level of competency comparable to dermatologists by CNN, which behaves equally well on both jobs as all verified experts. Gessert et al. (11) demonstrated a multiclass classification job utilizing an ensemble model created from Efficient Nets, SENet, and ResNeXt WSL on the ISIC 2019 dataset. The authors employed a cropping method on photographs to deal with the multiple models’ input resolutions. For unbalanced datasets, a loss-balancing method was also employed. Authors in Ref. (12) suggested a new CNN architecture for skin lesion categorization that consists of several tracts. A CNN has been retrained for multi-resolution input after it had been trained on a single resolution. Transfer learning is used to train seven classes of the HAM10000 dataset using a pre-trained MobileNet model. A categorical accuracy of 83.1% is reported, as well as precision, recall, and F1 scores of 89, 83, and 83%, respectively. (13) The importance of dermoscopy skin cancer images being classified as malignant or benign to detect melanoma was emphasized. The authors evaluated the ISIC archive dataset; the proposed solution achieved an accuracy of 81.3%, a precision of 79.74%, and a recall of 78.66% using transfer learning and the VGGNet convolutional neural network. This technique, however, was limited to a binary classification of skin cancer. When classifying skin cancer into three groups, Harangi et al. (14) looked at how an ensemble of deep CNNs may be utilized to increase the accuracy of individual models. The GoogleNet, AlexNet, ResNet, and VGGNet models’ respective accuracy rates were 84.2, 84.88, 82.88, and 81.38%. The best accuracy, 83.8%, was again reached by the combination of the GoogleNet, AlexNet, and VGGNet models. In addition, the recall rates for each of their models were 59.2, 51.8, 52.0, and 43.4%, respectively. Authors in Ref. (17) conducted a comprehensive analysis of seven distinct deep learning-based approaches for skin cancer. On the ISIC-2018 challenge dataset, experiments were conducted on neural networks such as PNASNet-5-Large, InceptionResNetV2, SENet154, and InceptionV4. The PNASNet-5-Large model has the highest accuracy at 76%. The authors in Ref. (15) obtained 99.03% accuracy, 99.81% recall, 98.7% precision, and a 99.25% F-score by proposing a deep pre-trained model of unclassified skin cancer images. The authors do not mention deleting some data from the dataset. The authors retrained the last layers of the proposed model on a small number of foot skin images. Furthermore, adding a new class to the eight classes makes it nine instead of eight, which makes the results comparison not applicable. A previous study in dermoscopic computer-aided classification has not only failed to obtain improved accuracy for skin cancer classification but also lacks generality. Unfortunately, much of the earlier research did not use large datasets, which are essential for deep learning models to perform well. In this study, the suggested strategy uses extremely accurate and efficient pre-trained models trained on a large ISIC 2019 dataset to obtain an exceptionally high accuracy for skin cancer classification.

### 2.1. Dataset

The most frequently used dataset in this field of research has been the ISIC 2019, which was employed.

The dataset is available at https://challenge.isic-archive.com/data#2016. ISIC 2019 dataset is one of the most difficult to classify into eight groups due to an uneven number of photographs in each class. The most difficult difficulty is detecting outliers or other “out of distribution” diagnosis confidence. The dataset was divided into three parts: training, validation, and testing, with the training portion comprising 80% of the dataset and the validation and testing portions each comprising 10%. The description of the dataset is summarized as follows:

The total number of images 25,331, dimension 256 × 256, color carding RGB, melanoma (MEL) 4,522, melanocytic nevus (NV) 12,875, basal cell carcinoma (BCC) 3,323, actinic keratosis (AK) 867, benign keratosis (BKL) 2,624, dermatofibroma (DF) 239, vascular lesion (VASC) 253, and squamous cell carcinoma (SCC) 628.

The validation/test set was used to validate the model on data it had never seen before, and the training set was used to train it.

### 2.2. Data augmentation

Deep learning models are data-hungry and generalize effectively when fed a large amount of data. Rotation, flip, random crop, modify brightness, adjust contrast, pixel jitter, aspect ratio, random shear, zoom, vertical and horizontal shift, and flip are utilized for data augmentation. Data augmentation is a method of artificially increasing the quantity of data available by adding slightly changed copies of existing training data rather than having to obtain new data. The training dataset size is intentionally increased, or the model is protected from over-fitting from the start, by either data warping or oversampling; in addition, it is used to improve the diversity of the data by slightly modifying copies of already existing data or creating synthetic data from existing data (23).

To augment it fairly, the ISIC 2019 dataset was tacitly divided into two sets, rich and poor. The rich set comprises “BCC,” “BKL,” “MEL,” and “NV” cancer classes with 23,344 instances in total. In contrast, the poor set involves “AK,” “DF,” “SCC,” and “VASC” classes with 1987 instances in total. Thus, the rich set occupies 92.16% of the dataset compared to the poor set which occupies 7.84%. Applying augmentation parameters to poor set classes to alleviate the imbalance in the training dataset while not applying augmentation to rich set classes. The poor set augmentation process is conducted on the fly (online augmentation) for the sake of saving resources and time for labeling at the expense of increasing training time.

## 3. Methods

The proposed model has two main experiments as follows: the first experiment used the augmented data for five different models for both Dag and Series Networks. A variety of topologies, including AlexNet, XceptionNet, DarkNet, DenseNet, and GoogleNet, were assessed.

Compared to each other, GoogleNet and DarkNet fared better than the rest of them. Applying transfer learning for each net will improve the accuracy as illustrated in Section 3.1. The second experiment applied to feature fusion for both GoogleNet and DarkNet through multi-SVM using ECOC is illustrated in Section 3.2.

### 3.1. Ranking trained models afterward transfer learning

To employ transfer learning, an algorithm is trained on one set of data and then applied to another set of data, which is referred to as a task linked to the original job.

To enhance generalization in another situation, domain adaptation and transfer learning are terms used to describe the phenomenon. Transfer learning is an exceptionally good technique in deep networks because of the immense resources needed and the vast quantity of pictures. Due to a tiny number of pictures, these data sets cannot be used to train deep neural networks from the beginning because of their lack of variety. This issue may have been solved through transfer learning (24).

**Figure 2:**
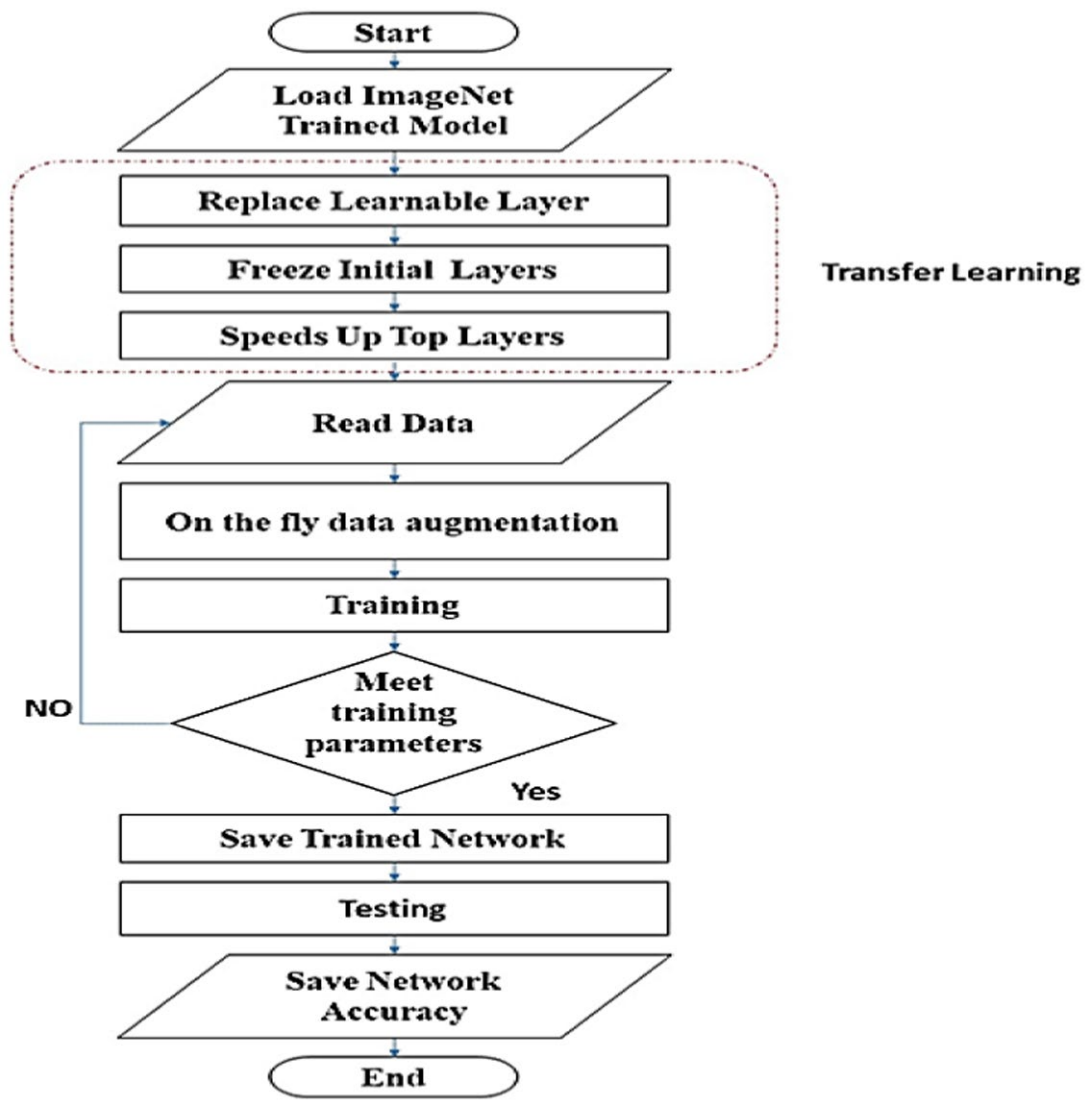
Flowchart of training and testing scheme to rank examined networks, according to their classification accuracy.

The imageNet (25) pre-trained models for classifying 1,000 objects are transferred to classify eight classes *via* transfer learning. There are three processes involved in achieving transfer learning. First, the last learnable layer for each net is altered one by one to classify eight skin classes. Second, it freezes the initial layers’ weights and biases to preserve their generalization extraction capability. Finally, by increasing the weights and biases learn rate factors, it speeds up the learning process for the deeper layers. Figure 2 illustrates the process of training and testing the picked-up networks to determine their classification accuracy.

### 3.2. Feature fusion methodology (FFM)

**Figure 3:**
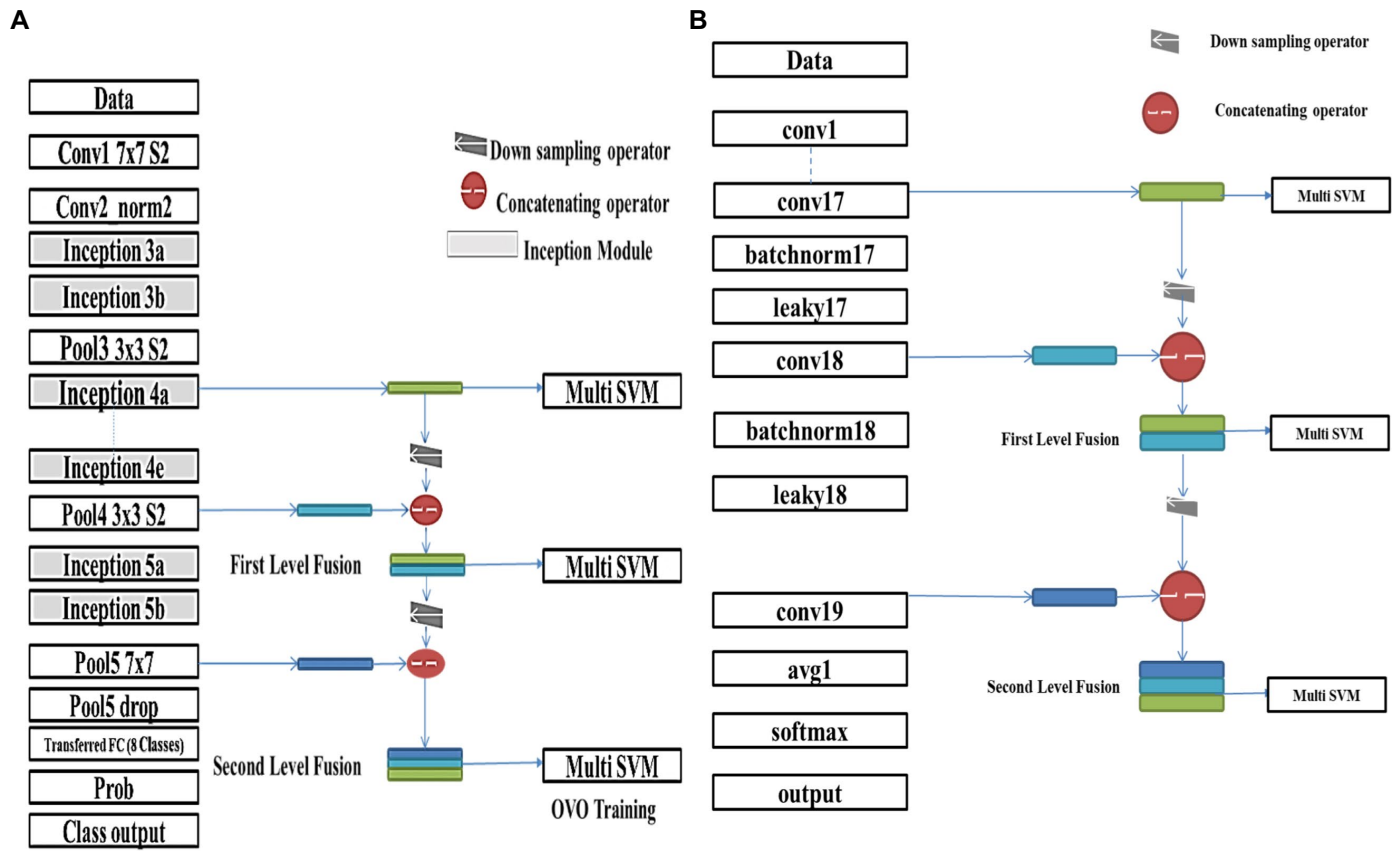
Proposed feature fusion scheme for **(A)** GoogleNet and **(B)** DarkNet.

The deepest (top) layer feature maps have the most abstract features that describe each class more semantically and contextually. While going backward for previous layers alleviates that description, it becomes higher resolution and may represent some abstracted features better. Applying the thought for enriching the feature descriptive supremacy *via* fusing different feature maps from various network layers will improve the accuracy for each net. In this study, the fusion is conducted for both trained networks (GoogleNet and DarkNet) at two levels, as follows:

1. GoogleNet’s inception4e is fused with *inception5a* feature map activations to form the first-level fusion. Furthermore, DarkNet’s *Conv17* is fused with *Conv18* feature map activations to spawn its first-level fusion.
2. GoogleNet’s resultant fused features are re-fused with *inception5b* activations, whereas the first-level fused DarkNet features are re-fused with *Conv19* activations, resulting in the second-level fused features for both networks. The activation of a layer is determined after training ends to obtain its feature maps, and the earlier feature maps are downsampled in a bilinear interpolation fashion to permit the concatenation process for fusion. The proposed feature fusion schemes are presented in Figures 3A,B for GoogleNet and DarkNet, respectively.

### 3.3. T-distributed stochastic neighbor embedding

For huge datasets, t-distributed stochastic neighbor embedding (t-SNE) has become the effective standard for visualizing high-dimensional datasets across a wide range of biomedical data.

**Figure 4:**
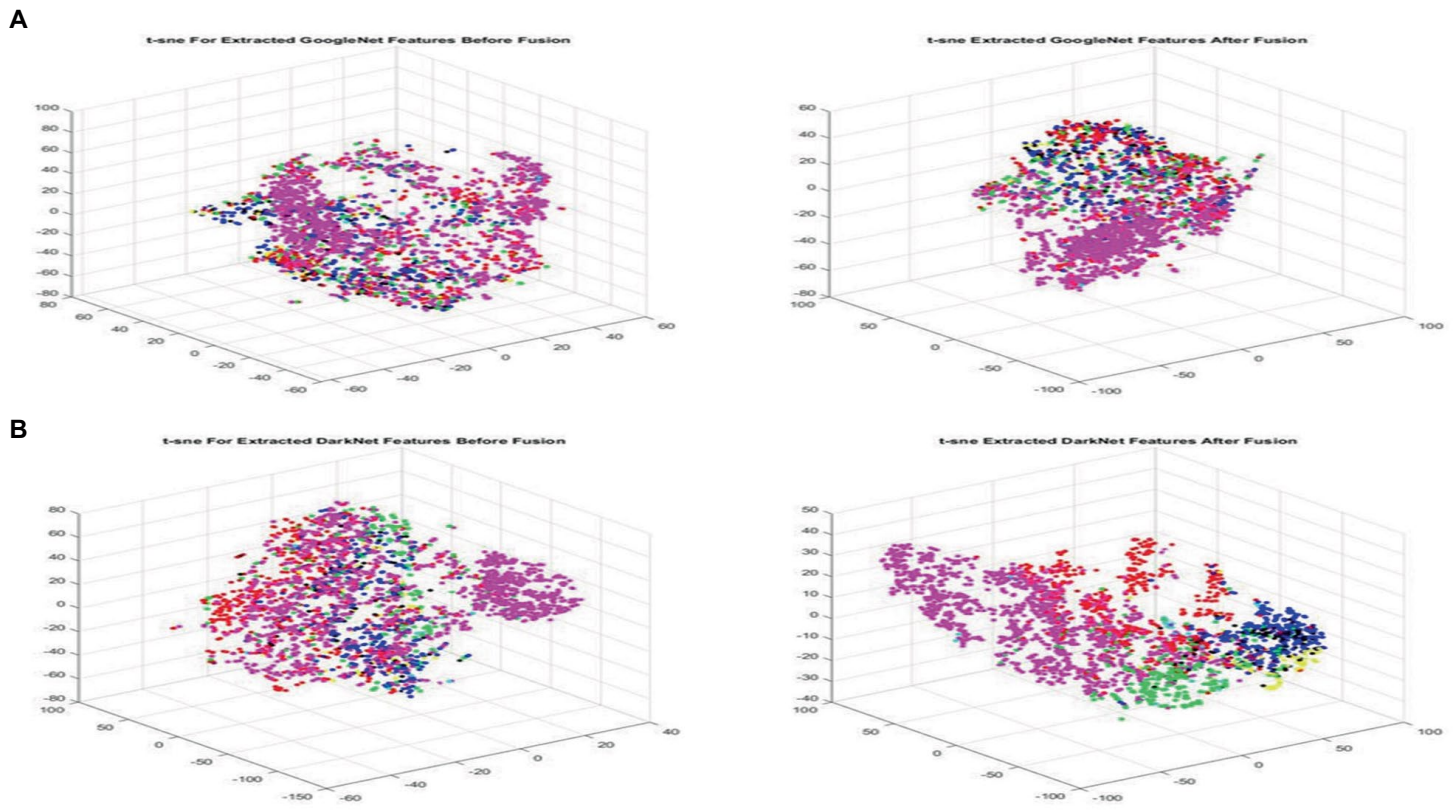
Visualizing extracted features before (left) and after (right) fusion using t-SNE for GoogleNet and DarkNet, respectively, best viewed in color. **(A)** GoogleNet and **(B)** DarkNet.

Using this technique to have a more clear visualization for each class will provide more clearance to researchers. T-SNE includes, but is not limited to, computer security, music analysis, cancer biology, and Bioinformatics. Similar to SNE, t-SNE chooses two different similarity measures between pairs of points for the high-dimensional information and the two-dimensional embedding. The goal of this step is to create a two-dimensional embedding that minimizes the KL divergence between the vector of similarities between points in pairs over the full dataset and the similarities between points in the encoding. The nonconvex optimization issue is solved using T-SNE using gradient descent with random initialization. Figures 4A,B visualizes the extracted features for both GoogleNet and DarkNet, respectively, before and after fusion using t-SNE. The fused features have a better similarity representation for instances belonging to the same class.

### 3.4. Error-correcting output codes (ECOC) model

An error-correcting output code (26) is utilized to simplify the multiclass classification task to binary ones. The simplification is usually conducted to train each learner in one of the two coding schemes which are one-versus-all (OvA) or one-versus-one (OvO). The desired training scheme is designed in a coding matrix (CM) that formulates how binary learner *L* considers class C in the training process.

The rows of the designed CM represent the classes while the columns are the learners. The filled values in each (*i,j*) matrix cell are altered between three values 0, 1, and − 1 representing that:

- An assigned value *1* marks all *jth* Class instances *Cj* as a positive class for the training of *ith* binary learner *Li*.
- An assigned value −1 marks all *jth* Class instances *Cj* as a negative class for the training of *ith* binary learner *Li*.
- An assigned value *0* discards all *jth* Class instances *Cj* for the training of ith binary learner *Li*.

Table 1 shows the true and false multi-SVM learners for eight classes. The true and false SVM learners for eight classes are shown in Table 2.

**Table 1:** Designed coding matrix for multi-SVM learners *Li* in OvO fashion for eight cancer classes *Cj.*

	*L*	*L*	*L*	*L*	*L*	*L*	*L*	*L*	*L*	*L*	*L*	*L*	*L*	*L*	*L*	*L*	*L*	*L*	*L*	*L*	*L*	*L*	*L*	*L*	*L*	*L*	*L*	*L*	
	*1*	*2*	*3*	*4*	*5*	*6*	*7*	*8*	*9*	*10*	*11*	*12*	*13*	*14*	*15*	*16*	*17*	*18*	*19*	*20*	*21*	*22*	*23*	*24*	*25*	*26*	*27*	*28*	
*C 1*	1	1	1	1	1	1	1	0	0	0	0	0	0	0	0	0	0	0	0	0	0	0	0	0	0	0	0	0	
*C 2*	−1	0	0	0	0	0	0	1	1	1	1	1	1	0	0	0	0	0	0	0	0	0	0	0	0	0	0	0	
*C 3*	0	−1	0	0	0	0	0	−1	0	0	0	0	0	1	1	1	1	1	0	0	0	0	0	0	0	0	0	0	
*C 4*	0	0	−1	0	0	0	0	0	−1	0	0	0	0	−1	0	0	0	0	1	1	1	1	0	0	0	0	0	0	
*C 5*	0	0	0	−1	0	0	0	0	0	−1	0	0	0	0	−1	0	0	0	−1	0	0	0	1	1	1	0	0	0	
*C 6*	0	0	0	0	−1	0	0	0	0	0	−1	0	0	0	0	−1	0	0	0	−1	0	0	−1	0	0	1	1	0	
*C 7*	0	0	0	0	0	−1	0	0	0	0	0	−1	0	0	0	0	−1	0	0	0	−1	0	0	−1	0	−1	0	1	
*C 8*	0	0	0	0	0	0	−1	0	0	0	0	0	−1	0	0	0	0	−1	0	0	0	−1	0	0	−1	0	−1	−1	

**Table 2:** Designed coding matrices for (a) multi-SVM true and (b) false learners. Where learner *li* is directed to deal with class *cj* as corresponding assigned values.

True	*L_1_*	*L_2_*	*L_3_*	*L_4_*	*L_5_*	*L_6_*	*L_7_*	*L_8_*		False	*L_1_*	*L_2_*	*L_3_*	*L_4_*	*L_5_*	*L_6_*	*L_7_*	*L_8_*
*C_1_*	1	−1	−1	−1	−1	−1	−1	−1		*C1*	−1	1	1	1	1	1	1	1
*C_2_*	−1	1	−1	−1	−1	−1	−1	−1		*C2*	1	−1	1	1	1	1	1	1
*C_3_*	−1	−1	1	−1	−1	−1	−1	−1		*C3*	1	1	−1	1	1	1	1	1
*C_4_*	−1	−1	−1	1	−1	−1	−1	−1		*C4*	1	1	1	−1	1	1	1	1
*C_5_*	−1	−1	−1	−1	1	−1	−1	−1		*C5*	1	1	1	1	−1	1	1	1
*C_6_*	−1	−1	−1	−1	−1	1	−1	−1		*C6*	1	1	1	1	1	−1	1	1
*C_7_*	−1	−1	−1	−1	−1	−1	1	−1		*C7*	1	1	1	1	1	1	−1	1
*C_8_*	−1	−1	−1	−1	−1	−1	−1	1		*C8*	1	1	1	1	1	1	1	-1
(a)										(b)								

These characteristics were classified using the multiclass SVM. To categorize fresh photographs, the multiclass SVM that was developed during the training phase may be used (test set). The ISIC 2019 test set was classified using the stored classifier, and the similarity score was calculated for each picture with various classifications.

### 3.5. Contradiction resolving using neutrosophic

**Figure 5:**
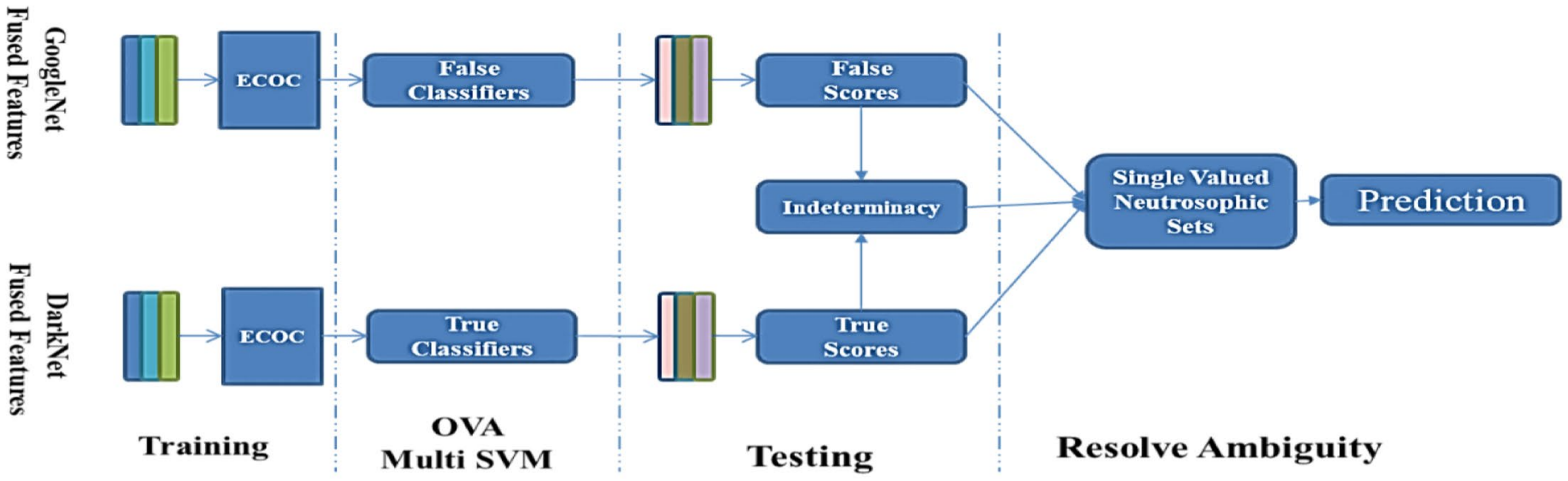
Proposed work’s overall scheme utilizes neutrosophic to resolve the feature fusion GoogleNet and DarkNet.

Figure 5 presents the feature fusion for GoogleNet and DarkNet and neutrosophic. Algorithm 1 states the main steps in the experiment as a pseudo-code.

### 3.6. Single-valued neutrosophic sets (SVNSs)

From a philosophical perspective, Ref. (27) extended the fuzzy set (28), the IFS, and the interval-valued IFS (29, 30) by introducing the neutrosophic set (31, 32). Authors in Ref. (33) used study data from Al-Bayrouni Hospital to categorize breast cancer. The used data were skewed and contains missing and anomalous numbers, so inverse Lagrangian interpolation and neutrosophic logic were used to rectify and analyze the data before formulating a two-kernel support vector machine algorithm. The proposed technique outperforms the traditional support vector machine algorithm since it is linearly orthogonal by neutrosophic logic and was trained on the data. This approach, which is based on inverse Lagrangian interpolation, correlates the inputs to determine how much each input belongs to each class. Authors in Ref. (34) include the idea of interval-valued Fermatean neutrosophic, Pythagorean neutrosophic, single-valued neutrosophic, and bipolar neutrosophic graphs. Many sorts of interval-valued Fermatean neutrosophic graphs as well as additional varieties of these graphs were described. This novel graph type was utilized in a situation involving decision-making. In addition, the interval-valued Fermatean neutrosophic number, interval-valued Fermatean triangle, and interval-valued Fermatean trapezoidal neutrosophic number were introduced. Authors in Ref. (35) analyzed interval-valued pentapartitioned neutrosophic graph features such as cut vertex, bridge, and degree, which are researched and thoroughly analyzed using relevant instances for making use of the suggested interval value. A decision-making technique called pentapartitioned neutrosophic graphs has been created and applied in a real-world scenario with numerical examples. In addition, the developed notions can be expanded to include isomorphic and regularity properties in the suggested graph topologies. Regular and irregular interval-valued pentapartitioned neutrosophic graphs, interval-valued pentapartitioned neutrosophic intersection graphs, interval-valued pentapartitioned neutrosophic hypergraphs, and other variations are all possible extensions of the interval-valued pentapartitioned neutrosophic graph. It is possible to model networks, telephony, image processing, computer networks, and expert systems using the interval-valued pentapartitioned neutrosophic graph.

Although new in principle, using neutrosophic sets in actual issues proved challenging, especially owing to the non-standard interval across which membership functions might take on values]-0,1+ [.

Three functions describe the membership of a neutrosophic set A in a universal set X: the truth function T(x), the indeterminacy function I(x), and the falsity function F(x). If X is a real standard or non-standard subset of]-0,1+ [, then T(x), I(x), and F(x) are all functions in X such that T(x):X→]-0,1+ [, I(x):X→]-0,1+ [, and F(x):X→]-0,1+ [. As a result, T(x), I(x), and F(x) may all add up to any value, therefore -0 ≤ supT(x) + supI(x) + supF(x) ≤ 3 + .

#### 3.6.1. Single-valued neutrosophic sets, DarkNet, and GoogleNet (SVNSs–DarkNet–GoogleNet)

SVNSs–DarkNet–GoogleNet is a suggested framework for dealing with uncertainty in skin cancer data that combines the approximate ideas provided by DarkNet and GoogleNet with the indeterminacy ideas of SVNSs. The SVNSs–DarkNet–GoogleNet is approachable postprocessing for uncertainty in DarkNet–GoogleNet model predictions that draw on neutrosophic ideas for multiple classes. The SVNSs–DarkNet–GoogleNet framework is used to assess the performance metrics, including accuracy, sensitivity, precision, and F1 score.

Algorithm 1 The pseudo-code of the proposed SVNSs.

**Table:** 

*Input:*	*ISIC 2019 dataset, Trained DarkNet, and GoogleNet*
*Output*	*SVNSs, framework performance measurements based on the estimated classes of instances.*
*Step 1:*	*Pre-process step* *1. construct fused features of all dataset instances according to the proposed FFM*
*Step 2:*	*Apply classification phase*
	*2. train true and false classifiers via ECOC and design coding matrices*
	*3. classify test set fused features for eight true classifiers* *4. for i = 1; i < = total number of test instances*
	*5. for t = 1; t < = total number of classes Cn*
	*6. compute similarity score SS (i, t)*
	*7. get the highest SS (i, t)*
	*9. output the class name (label)* *10. save true scores matrix T*
	*11. classify test set fused features for 8 false classifiers* *12. for i = 1; i < = total number of test instances*
	*13. for t = 1; t < = total number of classes n*
	*14. compute similarity score SS (i, t)*
	*15. get the lowest SS (i, t)*
	*16. output the corresponding class name (label)* *17. save false scores matrix F*
	*END*
*Step 3:*	*SVNSs characterization phase* *18. Construct indeterminacy I, code-word CW, and crisp CR matrices* *19. for i = 1; i < = total number of test instances* *20. for k = 1; k < = total number of classes n*
	*21. calculate indeterminacy membership value by* $I_{k}^{i}=1-|T_{k}^{i}-F_{k}^{i}|$
	*22. if* $T_{k}^{i}>F_{k}^{i}$ , $CW_{k}^{i}$ *= 1 else* $CW_{k}^{i}$ *=0* *23.* $CR_{k}^{i}=\left(\frac{2+T_{k}^{i}-I_{k}^{i}-F_{k}^{i}}{3}\right)$ *END* *END* *24. Resolving ambiguity* *25. for each code-word i in CW* *26. if only one-bit code-word (no ambiguity)*
	*27. if all code-word k of all classes output = 0, then corresponding* $\underset{k=1:n}{\max}I_{k}^{i}=1\;$ *and the rest = 0*
	*28. if two classes or more have bit-code = 1, then corresponding* $\;\underset{k=1:n}{\max}CR_{k}^{i}=1\;$ *and the rest = 0*
*Step 4:*	*Evaluation step*
	*1. find SVNSs class of unknown image*
	*2. construct a confusion matrix*
	*3. compute accuracy rate, precision, sensitivity, and f1-score*
	*Return SVNSs classes of an unknown image*
	*Return accuracy rate, precision, and f1-score for the SVNSs-model*

To forecast the scaling values of output classes, SVNSs–DarkNet–GoogleNet is constructed from two separate DarkNet–GoogleNet and they are trained using the same characteristics as input vectors. The DarkNet forecasts correct membership as true (T) decisions, whereas the GoogleNet forecasts incorrect ones as false (F). As shown in Algorithm 1, the conclusion of such classification is defined by SVNSs-TIF values, which are generated by the uncertainty border zone used to compute indeterminacy values (I).

To anticipate the opposite target value (code-word/on-hot-encoding), false GoogleNet is trained differently from true DarkNet. The number of distinct categories in the output is proportional to the length of the code-words. For example, if the true values are greater than the false values of the k^th bit of the codeword representing the k^th class is set to 1 and the rest of the bits are set to 0, then the k^th bit of the codeword representing the k^th The class will be set to 0, and the rest of the bits will be set to 1 if the false values are greater than the true values.

Binary predictions for multiple classifications in the SVNSs–DarkNet–GoogleNet model are very sensitive to the genuine membership code-word, as shown in Step 3 of the method shown in Algorithm 1. When a conflict arises between two possible outcomes, a codeword of 0 or several bits in the same codeword each equaling 1 in Step 3 (27–28) is employed to make a call.

If the expected value of the genuine DarkNet–GoogleNet is high, then the predicted value of the false membership DarkNet should be low, and vice versa. Due to their inconsistency, a zone of ambiguity has emerged. In line (1) of the SVNS definition, we see that the difference between the true and false membership values may be used as a rough approximation of the indeterminacy membership value. If there is not much of a disparity between them, then the uncertainty will be large, and vice versa.

## 4. Results

The training phase was conducted on an i7 10th generation machine @2.6GHz using NVIDIA GeForce GTX 1060 Ti GPU. An x64-bit MATLAB 2021-b was employed to perform the program. The performance of the proposed model was evaluated using four quantitative measures, accuracy, sensitivity, precision, and F1 score (36). These measures are computed as follows:


(1) $$\mathrm{Accuracy}=\frac{{\mathrm{tr}}_{\mathrm{pos}}+{\mathrm{tr}}_{\mathrm{neg}}}{{\mathrm{tr}}_{\mathrm{pos}}+{\mathrm{fls}}_{\mathrm{pos}}+{\mathrm{fls}}_{\mathrm{neg}}+{\mathrm{tr}}_{\mathrm{neg}}}$$

(2) $$\mathrm{Sensitivity}\left(\mathrm{Recal or true positive rate}\right)=\frac{{\mathrm{tr}}_{\mathrm{pos}}}{{\mathrm{tr}}_{\mathrm{pos}}+{\mathrm{fls}}_{\mathrm{neg}}}$$

(3) $$\mathrm{Precision}\left(\mathrm{positive predictive value}\right)=\frac{{\mathrm{tr}}_{\mathrm{pos}}}{{\mathrm{tr}}_{\mathrm{pos}}+{\mathrm{fls}}_{\mathrm{pos}}}$$

(4) $$F1\;\mathrm{score}=2\times \frac{\mathrm{Precision}\times \mathrm{Sensitivity}}{\mathrm{Precision}+\mathrm{Sensitivity}}$$

This is referred to as true positive ( $tr_{pso}$ ), false positive ( $fls_{pos}$ ), true negative ( $tr_{neg}$ ), and false negative ( $fls_{neg}$ ).

Unlike traditional approaches to updating parameters in gradient descent, SGD requires a single record at a time. However, because of its dependence on forward and backward propagation for each record, SGD is sluggish to converge. The road to a global minimum gets cluttered with noise. For the trained models that utilized an adaptive moment estimation (ADAM) solver, the training was conducted with an initial learning rate of 0.00003 and a square gradient decay factor of 0.99. While for stochastic gradient descent with momentum (SGDM) solver, the training was conducted with an initial learning rate of 0.00100 for the initial learning rate with a 0.1 Learning rate drop factor.

Table 3 illustrates the result of the first experiment, trained DAG and Series Networks Parameters and Accuracy in individual use to show the superior for each net, respectively.

**Table 3:** Trained network parameters and their classification accuracy.

Parameters	Input size	Mini-batch size	Solver	Number of iterations	Classification accuracy %
Network					
AlexNet (37)	[227,227,3]	227	ADAM	2,580	71.13
GoogleNet (38)	[224,224,3]	114	SGDM	2,900	77.41
DenseNet (39)	[224,224,3]	64	SGDM	3,043	73.44
XceptionNet (40)	[299,299,3]	20	SGDM	22,727	74.29
DarkNet (41)	[256,256,3]	114	ADAM	7,965	82.42

The easiest technique to enhance deep neural network efficiency is to increase the network capacity or depth. The depth corresponds to the number of network layers (levels). To train deeper models, a vast amount of labeled data was necessary. In this manner, there are two negatives to be considered. To begin, there are a lot of variables to consider.

When a small, labeled dataset is utilized for training, these values may lead to architectural overfitting. The second problem is that utilizing a network with several hidden layers increases the computing cost.

**Figure 6:**
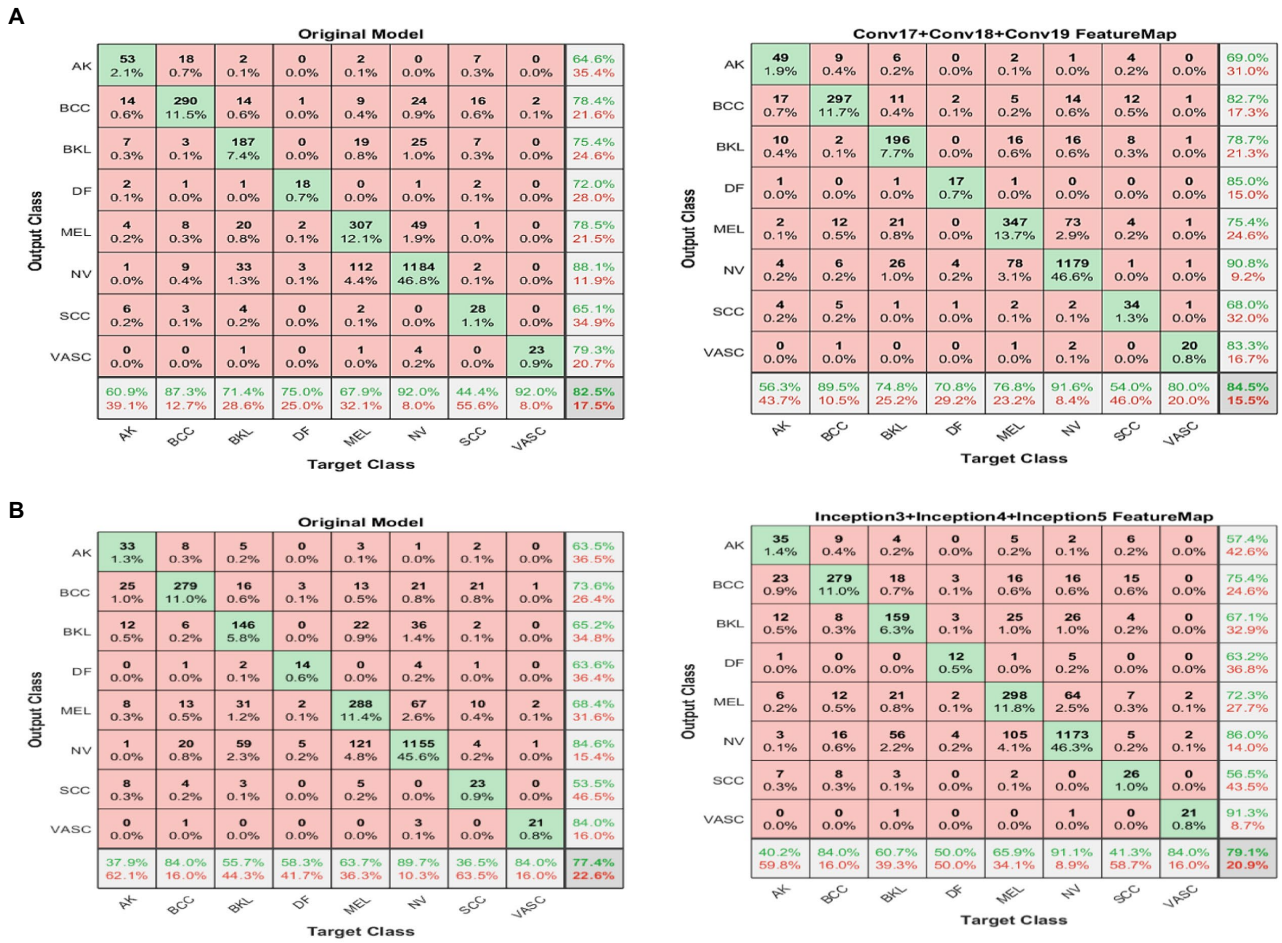
Confusion matrix (8 × 8) for the DarkNet and GoogleNet experiment (left) before and (right) after applying the proposed FFM. **(A)** GoogleNet and **(B)** DarkNet.

Figures 6A,B shows results for the second experiment, the confusion matrix (8×8) for the target class feature map for both DarkNet and GoogleNet, respectively.

An unfamiliar picture is one with a score of less than the value required for its classification as a similar image. This experiment used 25,331 photographs in total. The proposed model was trained and validated employing 80*%* of the ISIC 2019 dataset, which equates to 20,256 photographs, and 10*%* of the dataset, which equates to 2,531 images.

**Figure 7:**
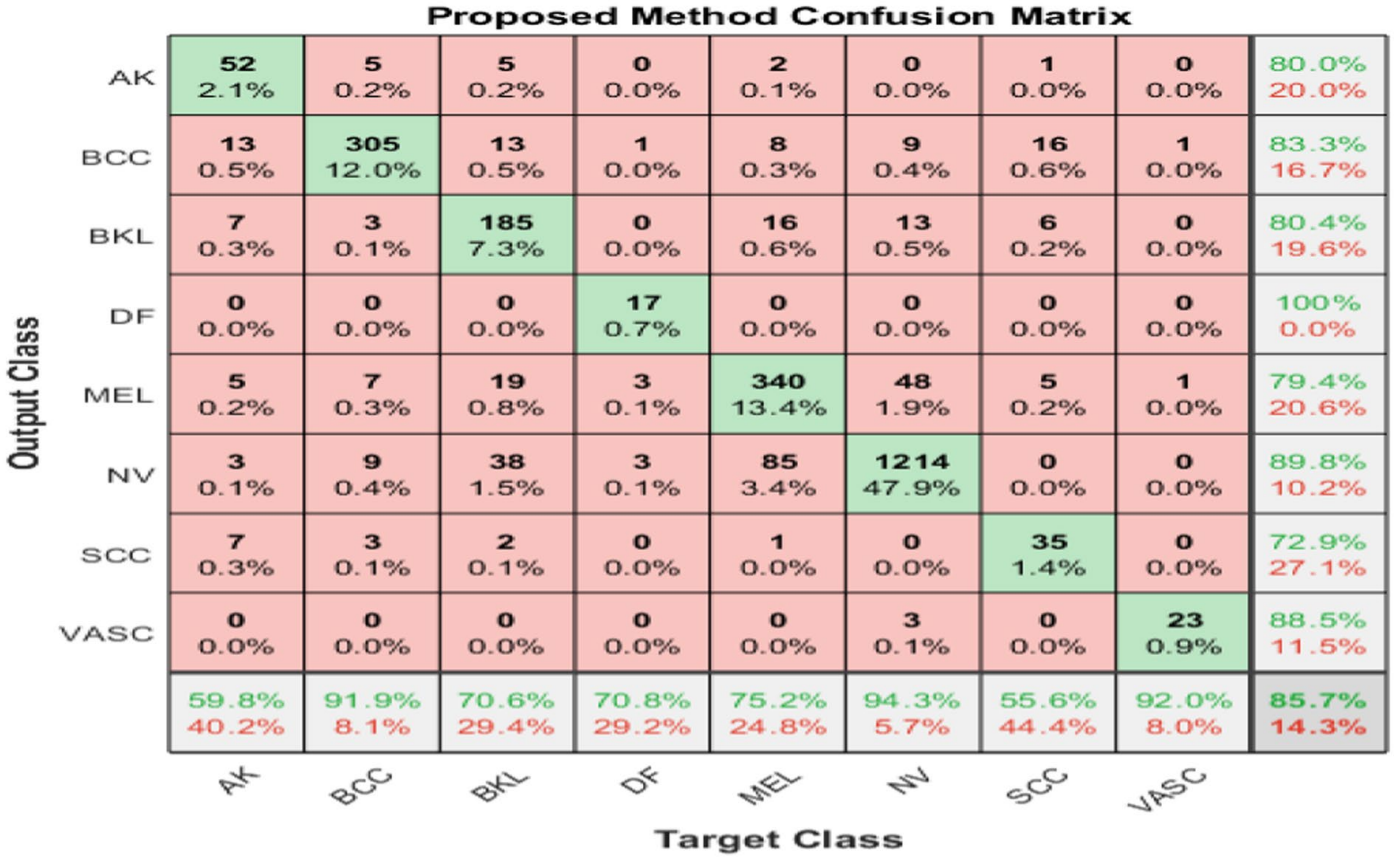
Resultant confusion matrix after contradiction resolving *via* SVNSs experiment.

Figure 7 shows the confusion matrix (8×8) for the target class feature map for combined fused and neutrosophic.

### 4.1. Overall results of (SVNSs–DarkNet–GoogleNet)

DarkNet–GoogleNet is done at this stage. True values are computed from the DarkNet, and false values are computed from GoogleNet. To make better-informed judgments in the uncertainty boundary zone, the SVNS characterization procedure calculates the indeterminacy values of the projected classes based on the SVNS definition. Algorithm 1 shows how the anticipated categorization of dataset samples is evaluated based on true (T), false (F), and indeterminacy (I) membership values, shows the code-word of all classes, and the crisp values of the eight test images.

Scaled values for T, I, and F membership are shown in Table 4. Using line (18) from Step 3 in Algorithm 1, we estimate the membership values of T and F for the two classes using DarkNet and GoogleNet and the membership values of I for the eight classes. Line (19) in Step 3 uses the code-words presented in Algorithm 1 to provide a binary classification of each class in the skin cancer dataset. The new examples of first and second image binary classification results are shown in Algorithm 1, where seven code-words are equivalent to zero, and one class is equivalent to one. From images 3 and 4, we have more than one class code-word that is equivalent to one, so we apply the equation of crisp value to select the max crisp value in Step 3 in line. (20) In Algorithm 1, from images 5 and 6 in Table 4, we have all code-words equivalent to zero, so we selected the max indeterminacy value from Step 3 in. line (21) In Algorithm 1, from Table 4 for eight images, SVNSs predicted eight true classes and zero false classes, DarkNet predicted only three true classes and five false classes, and GoogleNet predicted six true classes and two false classes. Table 5 shows the SVNS classification under eight classes. Figure 6 presents the feature fusion for GoogleNet and DarkNet and neutrosophic. Algorithm 1 states the main steps in the experiment as a pseudo-code.

**Table 4:** Single-valued neutrosophic sets.

Classes	SVNS	AK	BCC	BKL	DF	MEL	NV	SCC	VASC
Image 1	TRUE	6.27E-09	1.38E-07	4.45E-05	2.8E-06	0.007004	0.983134	2.04E-06	3.44E-06
	FALSE	0.999827	0.99999	0.998686	0.999999	0.974136	0.004948	0.999997	0.999897
	Code-Word	0	0	0	0	0	**1**	0	0
	Indeterminacy	0.000173	1.04E-05	0.001358	4.11E-06	0.032868	**0.021814**	5.02E-06	0.000107
	Crisp Value	0.333333	0.333333	0.333333	0.333333	0.333333	0.985457	0.333333	0.333333
Image 2	TRUE	1.5E-09	0.002268	1.14E-05	2.82E-07	0.991507	3.46E-07	4.74E-07	7.79E-07
	FALSE	0.999998	0.999967	0.998741	0.999854	0.032554	0.991417	0.999699	0.999431
	Code-Word	0	0	0	0	**1**	0	0	0
	Indeterminacy	2.39E-06	0.002301	0.001271	0.000146	**0.041047**	0.008583	0.000301	0.00057
	Crisp Value	0.333333	0.333333	0.333333	0.333333	0.972635	0.333333	0.333333	0.333333
Image 3	TRUE	2.79E-08	1.04E-07	1.85E-06	5.24E-05	0.91627	0.431186	2.52E-06	3.94E-08
	FALSE	0.999978	0.999988	0.998547	0.999833	0.686364	0.301565	0.99951	1
	Code-Word	0	0	0	0	**1**	**1**	0	0
	Indeterminacy	2.17E-05	1.25E-05	0.001455	0.000219	0.770094	0.870379	0.000492	3.94E-08
	Crisp Value	0.333333	0.333333	0.333333	0.333333	**0.486604**	0.419747	0.333333	0.333333
Image 4	TRUE	1.79E-07	3.5E-09	5.21E-08	4.74E-05	0.730593	0.750079	5.19E-07	3.58E-08
	FALSE	0.999598	0.998762	0.996136	0.999917	0.304397	0.431489	0.99994	0.999985
	Code-Word	0	0	0	0	1	1	0	0
	Indeterminacy	0.000402	0.001238	0.003864	0.00013	0.573804	0.68141	6.06E-05	1.47E-05
	Crisp Value	0.333333	0.333333	0.333333	0.333333	0.617464	0.545727	0.333333	0.333333
Image 5	TRUE	5.67E-07	0.00043	0.000133	3.69E-09	0.004654	0.858638	5.46E-05	9.5E-05
	FALSE	0.999527	0.99961	0.996572	0.999958	0.00835	0.97419	0.999923	0.998474
	Code-Word	**0**	**0**	**0**	**0**	**0**	**0**	**0**	**0**
	Indeterminacy	0.000473	0.00082	0.00356	4.22E-05	**0.996304**	0.884448	0.000131	0.001621
	Crisp Value	0.333333	0.333333	0.333333	0.333333	0.333333	0.333333	0.333333	0.333333
Image 6	TRUE	1.24E-09	0.21793	0.131226	0.002955	0.015759	6.38E-06	0.005419	5.97E-07
	FALSE	0.999643	0.996873	0.895743	0.997799	0.995986	0.934151	0.781645	1
	Code-Word	**0**	**0**	**0**	**0**	**0**	**0**	**0**	**0**
	Indeterminacy	0.000357	0.221057	**0.235483**	0.005155	0.019774	0.065856	0.223774	5.97E-07
	Crisp Value	0.333333	0.333333	0.333333	0.333333	0.333333	0.333333	0.333333	0.333333
Image 7	TRUE	2.88374E-05	4.33938E-07	0.13733686	0.00021989	0.03810337	0.41384637	0.00015349	5.45065E-06
	FALSE	0.99969565	0.99934214	0.42656824	0.94402712	0.97042959	0.76180088	0.99688428	0.99993538
	Code-Word	**0**	**0**	**0**	**0**	**0**	**0**	**0**	**0**
	Indeterminacy	0.00033317	0.00065829	**0.71076862**	0.05619277	0.06767378	0.65204548	0.00326921	7.00621E-05
	Crisp Value	0.33333333	0.33333333	0.33333333	0.33333333	0.33333333	0.33333333	0.33333333	0.33333333
Image 8	TRUE	1.66308E-06	0.81519597	1.80939E-09	2.78238E-10	0.96386611	3.09955E-07	6.69544E-06	9.52509E-07
	FALSE	0.99988746	0.03310882	0.98594981	0.99999880	0.77315056	0.99997746	0.99433112	0.99966990
	Code-Word	0	**1**	0	0	**1**	0	0	0
	Indeterminacy	0.00011419	0.21791284	0.01405018	1.19237E-06	0.80928444	2.28405E-05	0.00567557	0.00033104
	Crisp Value	0.33333333	**0.85472477**	0.33333333	0.33333333	0.46047703	0.33333333	0.33333333	0.33333333

The bold values are illustrate the value that the code will use to distinguish between the eight classes to determine one of them, either crisp value or indeterminacy.

**Table 5:** SVNSS classification of eight classes.

Image #	DarkNet	GoogleNet	SVNS	State True
Image 1	NV	NV	NV	NV
Image 2	MEL	MEL	MEL	MEL
Image 3	MEL	NV	MEL	MEL
Image 4	NV	MEL	MEL	MEL
Image 5	NV	MEL	MEL	MEL
Image 6	BCC	SCC	BKL	BKL
Image 7	NV	BKL	BKL	BKL
Image 8	MEL	BCC	BCC	BCC

Table 6 shows the overall results for (I) the original model and (II) FFM and (III) combined FFM with neutrosophic experiments. Table 7 shows the average performance to show the impact of the two experiments.

**Table 6:** Overall results of combined fused and neutrosophic experiments for eight classes.

Class			VASC	SCC	NV	MEL	DF	BKL	BCC	AKIEC
Metric/Model										
Precision (%)	GoogleNet	Original Model	84.0	53.5	84.6	68.4	63.6	65.2	73.6	63.5
		Second level Fusion	91.3	56.5	86.0	72.3	63.2	67.1	75.4	57.4
Sensitivity (%)		Original Model	84.0	36.5	89.7	63.7	58.3	55.7	84.0	37.9
		Second level Fusion	84.0	41.3	91.1	65.9	50.0	60.7	84.0	40.2
F1-score (%)		Original Model	91.2	69.1	85.5	77.1	77.6	76.4	82.6	76.8
		Second level Fusion	95.3	71.6	87.0	80.0	77.2	77.9	83.8	72.1
Precision (%)	DarkNet	Original Model	79.3	65.1	88.1	78.5	72.0	75.4	78.4	64.6
		Second level Fusion	83.3	68.0	90.8	75.4	85.0	78.7	82.7	69.0
Sensitivity (%)		Original Model	92.0	44.4	92.0	67.9	75.0	71.4	87.3	60.9
		Second level Fusion	80.0	54.0	91.6	76.8	70.8	74.8	89.5	56.3
F1-score (%)		Original Model	88.3	78.3	88.8	84.3	83.5	83.9	86.0	77.7
		Second level Fusion	90.8	80.4	90.9	82.6	91.7	86.2	88.9	80.9
SVNSs-DarkNet-GoogleNet		Precision (%)	88.5	72.9	89.8	78.9	100.0	80.0	83.6	78.5
		Sensitivity (%)	92.0	55.6	94.1	75.4	70.8	70.2	91.9	58.6
		F1-score (%)	84	93.8	83.8	90.7	85.0	99.9	86.9	89.6

**Table 7:** Overall average performance under the two experiments.

Experiments	Accuracy %	Sensitivity %	F1-score %	Precision%
GoogleNet model	77.4	63.7	79.5	69.5
Fused GoogleNet	79.1	64.7	80.6	71.1
DarkNet model	82.5	73.9	83.9	75.2
Fused Darknet	84.5	74.2	86.6	79.1
Proposed Work	85.74	76.1	89.6	84

## 5. Discussion

The purpose of this study is to create a diagnostic tool that divides skin lesion photographs into multiple classes. We rigorously evaluate a variety of DL models on the same dataset, the freely accessible ISIC 2019 database, to acquire a consistent set of measures for their performance because the training/testing dataset has an impact on the findings. The obvious imbalance in the training dataset affects how well the DL models perform; a set of spatial augmentation for the small instances classes was carried out tacitly as a remedy. The transfer learning is applied with the pre-trained GoogleNet, Xception, DarkNet, DenseNet, and AlexNet to exploit their efficient generalization and shorten training time. The trained models are ranked to pick the top two models for further improvements. A simple yet efficient feature fusion methodology is applied to boost the individual model’s performance. In addition, we show that using a neutrosophic environment to combine the boosted individuals maximizes classification performance, as evidenced by improvements in the collection of measurements (accuracy, precision, recall, and F-score).

The accuracy of the pre-trained models is listed in Table 3. We use the same procedure for each pre-trained model as shown in Figure 3. There are three processes involved in achieving transfer learning: altering the last learnable layer, freezing the initial layers, and accelerating the learning process for the deeper layers. The accuracy for the Xception, AlexNet, and DenseNet models stays the same and does not increase with the number of epochs approximately 0.74, 0.71, and 0.73 correspondingly as stated in Table 3.

After a few epochs, the loss function reaches a minimum and stays there, indicating that the model has stopped learning. Furthermore, as more epochs are added, both GoogleNet and DarkNet’s accuracy improves, yielding converged accuracy values of 0.77 and 0.82, respectively. Table 6 also includes the additional measures (precision, recall, and F-score). DarkNet, which has a value of 0.75, and GoogleNet, which has a value of 0.7, both produce the highest precision. DarkNet has the highest recall, followed by GoogleNet with values of 0.74 and 0.64, respectively. Finally, DarkNet has the greatest F-score when compared to GoogleNet, with a value of 0.84. These findings demonstrate that GoogleNet and DarkNet perform similarly on this database in terms of accuracy and F-score, with GoogleNet having a higher overall precision. As a result, DarkNet is the skin cancer detection model with the highest overall efficiency. Figure 4 is a qualitative visualization *via* the t-SNE to echo the proposed FMM efficiency.

The ECOC flexibility scheme eases implementing the proposed classification ensemble method as explained in Subsection 2.3.4. That flexibility appears obviously in the designed coding matrices in Table 2 which construct the true and false classifiers for DarkNet and GoogleNet, respectively. The disagreement between each true classifier and its opponent about an instance classification class creates ambiguity. The proposed scheme adopts the SVNSs to resolve that ambiguity to tilt the balance toward the correct skin class as listed in Algorithm 1. Moreover, Table 4 shows a practical application of Algorithm 1 on a sample from the test set with and without classification results agreement.

Table 8 recapitulates the comparative study between the proposed study and the recent other two related studies using the same dataset and the same number of classes.

**Table 8:** Comparison with the recent related study.

Model	Accuracy	Precision	Sensitivity	Year of study
EfficentNets (11)	63	–	73	Early 2020
DCNN (15)	81	77	74	2020
Proposed work	85.74	84	76.1	2023

## 6. Conclusion

Numerous studies have been conducted on the classification of skin cancer, but most of them were not successful in extending their research to the high-performance classification of various classes of skin cancer. The proposal investigates leveraging the classification accuracy in two successive stages: first, boosting the classification accuracy of the trained networks individually. A proposed feature fusion methodology is applied to enrich the extracted features’ descriptive power, which raises the accuracy by approximately 2%. The second stage explores how to combine the top two networks for further improvement. A set of true and false classifiers is built *via* the ECOC scheme, utilizing the fused features of the top two networks. Each true classifier and its opponent may disagree with the classification result, which establishes a contradiction. This contradiction is quantified by an indeterminacy set and resolved by the single-valued neutrosophic sets, which add 1.2% accuracy. The proposed study is generic and applicable to various classification tasks, and the extensive experiments conducted divulge its superiority and efficiency. In further research, the classification accuracy maybe improved using a preprocessing data purification technique to remove occlusions and hair from dataset instances.

## Data availability statement

The original contributions presented in the study are included in the article/supplementary material, further inquiries can be directed to the corresponding author.

## Author contributions

AA: conceptualization and writing—original draft. AA and AM: formal analysis and methodology. AA and NK: funding acquisition and resources. HM and AM: investigation. AM: software and visualization. AM, AA, and HM: validation. AA, AM, and NK: writing—review and editing. All authors have read and agreed to the published version of the manuscript.

## Conflict of interest

The authors declare that the research was conducted in the absence of any commercial or financial relationships that could be construed as a potential conflict of interest.

## Publisher’s note

All claims expressed in this article are solely those of the authors and do not necessarily represent those of their affiliated organizations, or those of the publisher, the editors and the reviewers. Any product that may be evaluated in this article, or claim that may be made by its manufacturer, is not guaranteed or endorsed by the publisher.

## Footnotes

^1^https://www.isic-archive.com, viewed on 6 October 2022.
